# Exome sequencing analysis of Japanese autism spectrum disorder case-control sample supports an increased burden of synaptic function-related genes

**DOI:** 10.1038/s41398-022-02033-6

**Published:** 2022-07-11

**Authors:** Hiroki Kimura, Masahiro Nakatochi, Branko Aleksic, James Guevara, Miho Toyama, Yu Hayashi, Hidekazu Kato, Itaru Kushima, Mako Morikawa, Kanako Ishizuka, Takashi Okada, Yoshinori Tsurusaki, Atsushi Fujita, Noriko Miyake, Tomoo Ogi, Atsushi Takata, Naomichi Matsumoto, Joseph Buxbaum, Norio Ozaki, Jonathan Sebat

**Affiliations:** 1 Department of Psychiatry, Nagoya University Graduate School of Medicine, Nagoya, Aichi Japan; 2 Public Health Informatics Unit, Department of Integrated Health Sciences, Nagoya University Graduate School of Medicine, Nagoya, Aichi Japan; 3 Department of Psychiatry, University of California San Diego, San Diego, CA USA; 4 Medical Genomics Center, Nagoya University Hospital, Nagoya, Aichi Japan; 5 Health Support Center, Nagoya Institute of Technology, Nagoya, Aichi Japan; 6 Department of Developmental Disorders, National Institute of Mental Health, National Center of Neurology and Psychiatry, Tokyo, Japan; 7 Department of Human Genetics, Yokohama City University Graduate School of Medicine, Yokohama, Kanagawa Japan; 8 Faculty of Nutritional Science, Sagami Women’s University, Sagamihara, Japan; 9 Department of Human Genetics, National Center for Global Health and Medicine, Tokyo, Japan; 10 Department of Genetics, Research Institute of Environmental Medicine, Nagoya University, Nagoya, Aichi Japan; 11 Department of Human Genetics and Molecular Biology, Nagoya University Graduate School of Medicine, Nagoya, Aichi Japan; 12 Laboratory for Molecular Pathology of Psychiatric Disorders, RIKEN Center for Brain Science, Wako, Saitama Japan; 13 Department of Psychiatry, Mount Sinai University, New York, NY USA; 14 Institute for Glyco-core Research (iGCORE), Nagoya University, Chikusa-ku, Nagoya, Japan

**Keywords:** Clinical genetics, Molecular neuroscience

## Abstract

Autism spectrum disorder (ASD) is a highly heritable, complex disorder in which rare variants contribute significantly to disease risk. Although many genes have been associated with ASD, there have been few genetic studies of ASD in the Japanese population. In whole exomes from a Japanese ASD sample of 309 cases and 299 controls, rare variants were associated with ASD within specific neurodevelopmental gene sets, including highly constrained genes, fragile X mental retardation protein target genes, and genes involved in synaptic function, with the strongest enrichment in trans-synaptic signaling (*p* = 4.4 × 10^−4^, *Q*-value = 0.06). In particular, we strengthen the evidence regarding the role of *ABCA13*, a synaptic function-related gene, in Japanese ASD. The overall results of this case-control exome study showed that rare variants related to synaptic function are associated with ASD susceptibility in the Japanese population.

## Introduction

Autism spectrum disorder (ASD) is a neurodevelopmental disorder characterized by deficits in social interactions and repetitive behaviors manifesting in early childhood [1]. Little is known regarding the pathogenesis of ASD, and current therapies such as pharmacotherapy and psychosocial interventions often only treat symptoms and are therefore insufficient for most patients with ASD. Additional research to develop new therapies by elucidating the pathophysiology of ASD is thus needed.

ASD is highly heterogeneous, with an estimated heritability as high as 80% [2]. Recent large-scale genetic analyses have revealed that rare (minor allele frequency < 1%) variants in which the major contributors are rare single-nucleotide variants (SNVs) that disrupt gene function or rare copy number variations (CNVs) detected by whole-genome/-exome sequencing (WGS/WES) [3, 4] could have large effect sizes. In terms of high effect size and the possibility of biological functional validation of rare variants, characterization of these variants offers more promise as a means of elucidating the pathophysiology of ASD and facilitating identification of novel drug targets [5] than characterization of common (minor allele frequency >5%) single-nucleotide polymorphisms (SNPs) identified by genome-wide association analyses [6].

Much of the gene discovery in ASD research in sequencing studies has focused on *de novo* variants [7, 8] and rare inherited variants discovered through family analyses [9]. However, one of the limitations of family studies is the small sample size resulting from limited access to family samples. Therefore, more recent research has also focused on case-control studies with larger sample sizes than family studies, and these analyses have demonstrated that rare variants prioritized by effect on protein function and frequency in public databases are enriched in various neurodevelopmental disorders, such as schizophrenia [10], ASD [11], and epilepsy [12]. However, these findings have primarily been derived from samples of European ancestry. Recent trans-ethnic analyses have examined commonalities and differences in the pathogenicity of neuropsychiatric disorders [13]. A previous WES study of Japanese ASD focusing only on *de novo* variants indicated common pathogenesis with samples of European ancestry [14]. However, there are no ASD case-control WES analyses focusing on the Japanese ASD population. By performing a case-control study, we could capture a broader range of rare variants, including rare inherited variants and *de novo* rare variants, compared with a previous study of rare *de novo* variants in trios [14]. Therefore, in this study, we performed a Japanese ASD case-control WES analysis to identify genes or genes sets associated with Japanese ASD pathobiology to facilitate the identification of novel drug targets.

Through this study, we found that rare variants in synaptic function-related genes are associated with susceptibility to ASD in the Japanese population.

## Methods

### Sample information

The case-control sample set used in this study included 309 Japanese ASD patients (mean age ± SD = 20 ± 11.1 years, proportion of males = 0.74) and 299 Japanese healthy control (HC) subjects (mean age ± SD = 38 ± 13.9 years, proportion of males = 0.70). All cases were included if they met the criteria for ASD in the Diagnostic and Statistical Manual of Mental Disorders, Fifth Edition. In the majority of ASD cases, diagnostic and screening instruments were used to evaluate ASD-related behaviors and symptoms: Autism Diagnostic Interview-Revised (ADI-R) [15], Autism Diagnostic Observation Schedule (ADOS) [16], Autism spectrum quotient [17], and Social Responsiveness Scale [18]. In addition, the patients’ capacity to consent was confirmed by a family member when needed. Controls were selected from the general population and had no history of mental disorders based upon responses to questionnaires or self-reporting. The vast majority of subjects were recruited from the central part of Honshu Island, the largest island of Japan. Written informed consent was obtained from all participants. The Ethics Committees of the Nagoya University Graduate School of Medicine and associated institutes and hospitals approved this study.

### WES and data processing

Genomic DNA was extracted from whole blood or saliva using a Qiagen QIAamp DNA blood kit or tissue kit (Qiagen, Hilden, Germany). Raw WES data were generated and processed to BAM files at three sites (Table S1): The Broad Institute (ASD = 256, HC = 299) [11] using Illumina HiSeq sequencers and an Illumina Nextera exome capture kit; Yokohama City University (ASD = 51) [14] using Illumina HiSeq with Agilent Sure Select v5; and Nagoya University (ASD = 2) using Illumina HiSeq with Agilent Sure Select v5. Detailed descriptions of each sequencing method are presented elsewhere [11, 14, 19]. Each sample’s sequencing reads were aligned onto the human genome build 37 (GRCh37/hg19) and then aggregated into a BAM file. Genomic variant call format (gVCF) files were generated using Haplotype Caller, version 4.1, in the Genome Analysis Toolkit (GATK) [20]. SNVs and insertions/deletions (indels) were jointly called across all samples (ASD = 309, HC = 299) using the GenotypeGVCF function.

### Data set quality check (QC)

A schematic illustration of the filtering step is presented in Fig. S1. Variant call accuracy was evaluated using the GATK variant quality score recalibration (VQSR) approach. For the QC, we firstly excluded variants that failed the VQSR, variants in low-complexity regions [21], and mitochondrial DNA regions. We picked variants in the common regions sequenced by each platform used in this study (Table S1). For individual-level genotype QC, genotype calls with read depth < 10, genotype quality < 25, and allele balance < 30 were masked using the variantFiltration function of GATK to enable the -setFilteredGtToNoCall option and then excluded.

As a sample QC, we excluded samples meeting the following conditions: (1) samples from the same family (relatedness_Phi ≥ 0.1 after analysis of relatedness [22]), (2) duplicate samples, and (3) low-quality samples (call rate ≥ mean – 3 SD). The sex of the individuals was confirmed using the -check-sex ycount function of PLINK. We then filtered variants with a call rate <70% across all samples. Principal component analysis (PCA) was performed to exclude population outliers using SMARTPCA based on a linkage disequilibrium (LD)-pruned set of 33,566 SNPs obtained by removing large-scale high-LD regions or SNPs with a genotype call rate <98%, minor allele frequency (MAF) < 0.01, or Hardy-Weinberg equilibrium (*P* < 1 × 10^−6^). LD pruning was performed using the PLINK option ‘--indep-pairwise 50 5 0.2’. A population outlier was detected and excluded from further analyses. Furthermore, PCA using the 1000 Genomes Project reference panel (phase 3) detected no subject with probable ancestry outside the East Asian population. The results of the PCA are shown in Fig. S2. Finally, 301 ASD patients and 296 HCs were included for further analyses.

### Variant prioritization

For functional annotation of variants, we used ANNOVAR [23] with the RefSeq database. First, we used the following procedure to analyze only autosomal rare variants. Variants with a MAF > 5.0 × 10^−4^ were excluded using the following public database: The Genome Aggregation Database (gnomAD V2.1.1) and Japanese Multi Omics Reference Panel (jMorp ToMMo 8.3 kJPN v20200831) (https://jmorp.megabank.tohoku.ac.jp/202102/). Furthermore, variants with a MAF > 5.0 × 10^−3^ in our case-control data were excluded. The overall number of rare variants for each sample was calculated and used as a confounding variable in the subsequent burden test. Each individual’s overall number of detected rare variants is shown in Fig. S3.

To prioritize variants for ASD pathophysiology, we picked likely loss of function (LoF) variants (startloss, stopgain, stoploss, frameshift deletion, frameshift insertion, canonical splicing site variation) and putative deleterious missense variants (D-mis) defined according to the following conditions: Combined Annotation-Dependent Depletion (CADD) score ≥ 30 and deleterious prediction by Polyphen-2 [24] and SIFT [25]. These prioritized variant sets were then used as the starting point for rare variant case-control association testing.

To further prioritize LoF variants, we picked LoF variants in constrained genes evaluated based on ExAC pLI scores [26] (pLI > 0.5), available online (https://gnomad.broadinstitute.org/downloads), and picked LoF variants in highly constrained genes (pLI > 0.9). We used these thresholds according to the largest ASD exome study so far [11]. In the study, protein truncating variants in high constraint genes such as pLI > 0.995, pLI > 0.9, and pLI > 0.5 were more enriched in ASD cases than controls, whereas the lowest tier (pLI < 0.5) showed no enrichment.

### Burden tests with prioritized rare variants

To determine whether ASD was associated with an increased number of prioritized rare variants in autosomal chromosomes, we performed rare variant case-control association tests based on the prioritized rare variants using the burden test with possible confounding variables, including each individual’s overall number of detected rare variants, sex, and the first 10 principal components estimated from the PCA. Burden tests are more powerful when most variants in a region are causal and the effects are in the same direction. The burden test was performed using the SKATBinary function with the option of method = ”Burden” in SKAT of the R package [27]. *P*-values < 0.05 were considered to indicate a nominal significant association.

### Gene set-based burden tests

We performed gene set–based burden tests to compare the proportions of cases and controls carrying one or more damaging rare variant (LoF and D-mis variants). We tested for associations within LoF and D-mis for following four gene sets: (1) fragile X mental retardation protein (FMRP) target genes from Table S2A of Darnell et al. [28]; (2) synaptic genes registered in the SynGO database, focusing on synaptic function and/or localization based on published, expert-curated evidence [29] (https://www.syngoportal.org); (3) genes encoding chromatin modifiers from Iossifov et al. [7]; and (4) ASD-related genes that are syndromic or that score 1, 2, or 3 in the SFARI database (01-13-2021_release) (https://gene.sfari.org). Burden tests were performed as described in the “Burden tests with prioritized rare variants” section. To correct multiple comparisons, *Q*-values derived via the Benjamin-Hochberg procedure [30] were calculated. The significance level was set at a *Q*-value of <0.1.

### Burden test with GO terms focusing on synaptic function

To identify GO terms related to synaptic functions that could be linked to ASD pathogenesis, we performed a burden test with prioritized LoF and D-mis variants using GO terms focusing on synaptic function, established as a SynGO analysis comprising 87 synaptic locations and 179 synaptic processes [29]. The burden test was performed as described in the “Burden tests with prioritized rare variants” section. To correct multiple comparisons, *Q-*values derived via the Benjamin–Hochberg procedure [30] were calculated. The significance level was set at a *Q*-value of <0.1. Visualization of clusters of significantly enriched SynGO terms was performed using Custom color-coding of SynGO ontologies (https://www.syngoportal.org/plotter.html).

### Genome-wide gene-based burden test

To identify candidate genes associated with ASD, we performed a genome-wide, gene-based rare variant burden test with prioritized LoF and D-mis variants. The burden test was performed as described in the “Burden tests with prioritized rare variants” section. To correct multiple comparisons, *Q*-values derived via the Benjamin–Hochberg procedure [30] were calculated. The significance level was set at a *Q*-value of <0.1.

### Expression analysis

We performed specific expression analysis (SEA) with human transcriptomic data from the BrainSpan [31] collection to identify particular human brain regions and/or developmental windows potentially related to ASD pathophysiology along with candidate genes identified in ASD patients in this study. Furthermore, to identify candidate brain cell populations likely to be disrupted across a set of ASD patients, we also performed cell type-specific expression analysis (CSEA) [32] of candidate genes identified in ASD patients. For each cell type or brain region, transcripts specifically expressed or enriched were identified at specificity index (pSI) thresholds of varying stringency [32] (e.g., pSI < 0.01 would identify a greater number of relatively enriched transcripts, whereas pSI < 0.0001 would identify relatively specific subsets). These analyses were performed using the server in the Dougherty lab (http://genetics.wustl.edu/jdlab/). Lists of candidate genes that overlapped with lists of transcripts enriched in a particular cell type or brain region were finalized using Fisher’s exact test with Benjamini–Hochberg correction. The significance level was set at *Q*-value < 0.1.

## Results

### Rare variant association analysis with overall prioritized variants

**Fig. 1:**
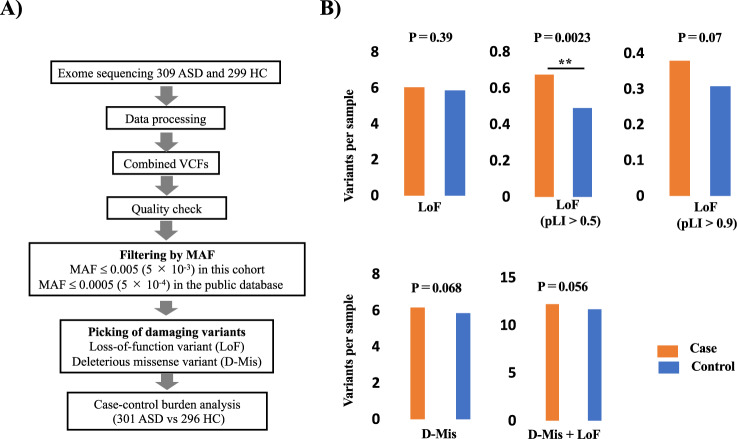
Results of rare variant association analyses using the burden test. **A** The primary filtering step used in this study. After sample and genotype quality checking, we picked LoF and D-Mis variants with MAF ≤ 5.0 × 10^−3^ in this cohort and ≤ 5.0 × 10^−^^4^ in the public databases for the subsequent case-control burden analysis. During the filtering step, 8 ASD and 3 HC samples were excluded (Fig. S1). **B** Burden tests were performed under each of the following conditions: LoF variants, LoF variants in genes with pLI score > 0.5, LoF variants in genes with pLI score > 0.9, D-mis variants, and D-mis + LoF variants. Although we did not detect any significant associations for overall LoF variants, LoF variants in genes with pLI > 0.5 were enriched in ASD cases. ** Indicates a nominal significant association (uncorrected *P*-value < 0.01).

After filtering WES data based on sample and genotype quality, we performed a rare variant burden analysis with prioritized rare variants (Fig. 1A) from 301 ASD patients and 296 HCs. The detailed filtering step is shown in Fig. S1.

Although the burden test (Fig. 1B) with overall LoF variants and D-Mis variants did not detect a significant association with ASD, LoF variants in genes with a pLI score >0.5 were significantly enriched in ASD patients (*P*-value = 0.0023) (Fig. 1B). LoF variants in genes with pLI > 0.9 (*P*-value = 0.07), D-Mis variants (*P*-value = 0.068), and D-Mis + LGD variants (*P*-value = 0.056) were not significant, but the same tendency as the LoF variants in genes with pLI > 0.5.

### Association analysis with gene sets related to ASD pathophysiology

**Fig. 2:**
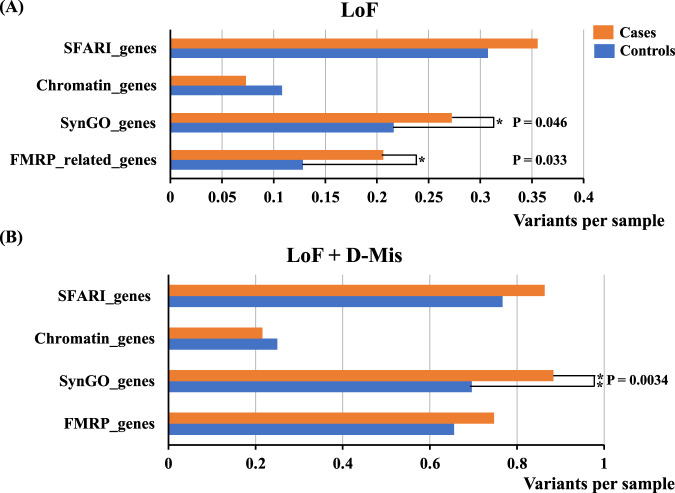
Results of burden tests using gene sets related to ASD pathophysiology. For the gene set burden tests, we used four gene sets: (1) FMRP target genes (FMRP_genes), (2) genes registered in the SynGO database (SynGO_genes), (3) genes encoding chromatin modifiers (Chromatin genes), (4) genes registered in the SFARI database (SFARI_genes). **A** The number of variants per sample for each gene set about LoF variants. **B** The number of variants per sample for each gene set about LoF + D-Mis variants. The LoF/LoF + D-Mis variants in the SynGO_genes were significantly enriched in ASD samples. The LoF variants in the FMRP_genes were significantly enriched in the ASD cases. The enrichment of the Chromatin_genes and SFARI_genes is not detected. An asterisk symbol indicates a nominal significant association (*P*-value < 0.05). Double asterisks indicate a nominal significant association (*P*-value < 0.01).

In addition to comparing the overall number of LoF and D-Mis variants, we performed a gene set burden analysis to elucidate the pathophysiology of ASD with four publicly available sets of genes that have been implicated in ASD susceptibility [7, 28, 29, 33]. We demonstrated that LoF variants in FMRP target genes and genes registered in SynGO were significantly enriched in the ASD samples (Fig. 2A). Furthermore, LoF + D-Mis variants in genes registered in SynGO were also enriched in the ASD (Fig. 2B).

### Burden test using gene sets related to synaptic function

**Fig. 3:**
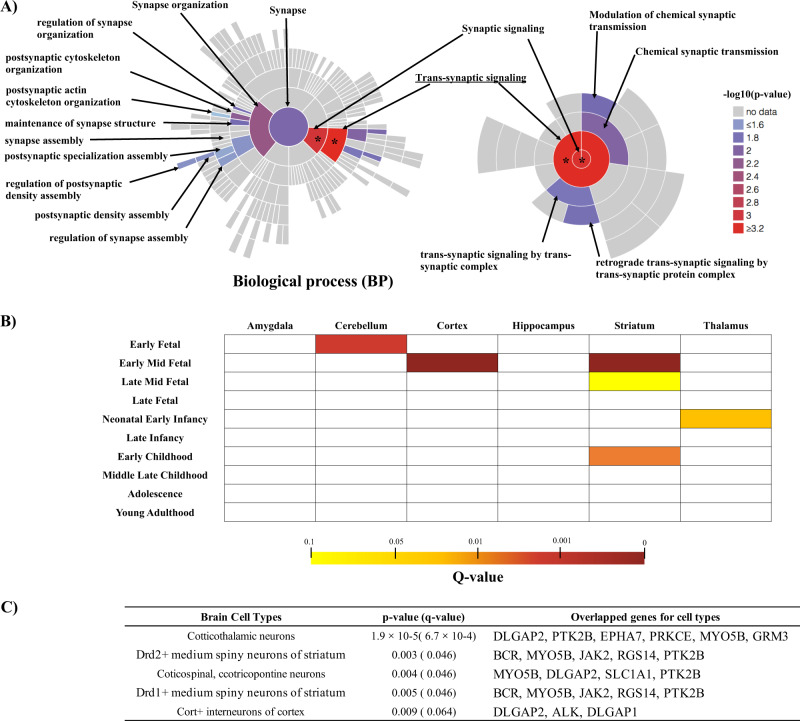
Detailed description of synaptic signaling–related genes in ASD patients in this study. **A** Visualization of the results of burden tests of nominal significant association performed using Custom color-coding of SynGO ontologies (https://www.syngoportal.org/plotter.html). All biological process (BP)-related terms populated with gene annotations in SynGO were plotted in a circular fashion, with the highest hierarchical term (synapse) in the center and each layer of subclasses in outward concentric rings (left panel). We colored ontology terms with *P*-values < 0.05 from the results of burden tests with gene set in SynGO (Table 1). We then visualized GO terms focusing on “synaptic signaling (GO:0099536)” that showed a significant association with ASD (right panel). Under “synaptic signaling”, trans-synaptic signaling (GO:0099537) showed the most significant association with ASD. Among subclasses of trans-synaptic signaling, several GO terms, such as chemical signal transmission (GO: 0007268), showed a nominally significant association with ASD. * Indicates a significant association considering multiple comparison (*Q*-value < 0.1). **B** Results of specific expression analysis (SEA) of genes among the prioritized variants of trans-synaptic signaling genes across brain regions and development. SEA revealed that genes in trans-synaptic signaling (GO: 0099537) detected in ASD cases were enriched during the early mid-fetal period in the cortex and striatum. Color bar shows *Q*-values. **C** Results of cell type-specific expression analysis (CSEA) with *Q*-value < 0.1 based on genes among the prioritized variants of trans-synaptic signaling genes. CSEA revealed that genes in trans-synaptic signaling detected in ASD cases were enriched in corticothalamic neurons and medium spiny neurons in the striatum.

To define what aspects of synapse function could be linked to ASD pathogenesis, we performed a burden test of LoF + D-Mis variants with GO terms focusing on synaptic function, established as a SynGO analysis comprising 87 synaptic locations and 179 synaptic processes [29]. The results of burden tests with nominal significant association are described in Table 1 and visualized in Figs. 3A and S4. In biological process (BP) category from SynGO terms, we found that trans-synaptic signaling (GO:0099537) showed a most significant association (*P* = 4.4 × 10^−4^, *Q*-value = 0.060) with ASD in this cohort. Synapse organization (GO: 0050808) also showed a nominally association (*P* = 4.9 × 10^−3^, *Q*-value = 0.21). In cellular component (CC) category from SynGO terms, post-synapse (*P* = 0.014, *Q*-value = 0.21) and post-synaptic density, intracellular component (*P* = 0.018, *Q*-value = 0.20) (a subclass of post-synapse), showed a nominally significant association with ASD (Fig. S4).

**Table 1:** Results of burden test with gene set in SynGO.

GO_terms	Ontology domain	Number of genes	MAC_Burden	Mean_case	Mean_ctrl	*P*-value	*Q*-value
Trans-synaptic signaling (GO:0099537)	BPa	185	84	0.19	0.09	0.00044	0.060
Synaptic signaling (GO:0099536)	BP	193	86	0.19	0.10	0.00072	0.060
Synapse organization (GO:0050808)	BP	306	144	0.30	0.18	0.0049	0.21
Postsynaptic cytoskeleton organization (GO:0099188)	BP	26	20	0.056	0.01	0.0070	0.21
Chemical synaptic transmission (GO:0007268)	BP	160	63	0.13	0.08	0.0098	0.21
Process in the synapse	BP	879	355	0.67	0.52	0.012	0.21
Modulation of chemical synaptic transmission (GO:0050804)	BP	90	33	0.073	0.04	0.012	0.21
Regulation of synapse organization (GO:0050807)	BP	29	13	0.037	0.01	0.014	0.21
Postsynapse (GO:0098794)	CC	624	232	0.45	0.33	0.014	0.21
Maintenance of synapse structure (GO:0099558)	BP	18	13	0.037	0.0068	0.015	0.21
Retrograde trans-synaptic signaling by trans-synaptic protein complex (GO:0098942)	BP	7	13	0.037	0.0068	0.015	0.21
Trans-synaptic signaling by trans-synaptic complex (GO:0099545)	BP	12	17	0.047	0.010	0.017	0.21
Postsynaptic density, intracellular component (GO:0099092)	CC	42	14	0.040	0.0068	0.018	0.21
Postsynaptic density assembly (GO:0097107)	BP	19	9	0.027	0.0034	0.023	0.23
Regulation of postsynaptic density assembly (GO:0099151)	BP	14	9	0.027	0.0034	0.023	0.23
Synapse assembly (GO:0007416)	BP	93	42	0.10	0.041	0.024	0.23
Regulation of synapse assembly (GO:0051963)	BP	50	22	0.056	0.017	0.026	0.23
Synapse (GO:0045202)	CC	1089	419	0.77	0.632	0.027	0.23
Postsynaptic specialization assembly (GO:0098698)	BP	32	17	0.043	0.014	0.030	0.25
Postsynaptic actin cytoskeleton organization (GO:0098974)	BP	22	13	0.037	0.0068	0.041	0.32

Note. *BP* biological process, *CC* cellular component, *Number of Genes* number of genes in each GO_term, *MAC_Burden* number of allele counts used for the burden analysis, *mean_case* mean number of variants in one case, *mean_ctrl* mean number of variants in one healthy control.

To identify particular human brain regions and/or developmental windows potentially related to ASD pathophysiology, we performed a SEA using human transcriptome data from the BrainSpan collection [31] and demonstrated that genes in variants in trans-synaptic signaling (GO:0099537) detected in ASD patients were enriched during the early mid-fetal period in the cortex (*P* = 3.0 × 10^−4^) and striatum (*P* = 3.0 × 10^−4^) (Fig. 3B and Table S2). Furthermore, CSEA revealed that genes in variants in trans-synaptic signaling (GO:0099537) detected in ASD patients were enriched in corticothalamic neurons and striatum medium spiny neurons (Fig. 3C and Table S3).

### Genome-wide gene-based burden test

As indicated by the possible overall enrichment of prioritized rare variants in ASD patients (Fig. 1), we performed a genome-wide gene-based burden test with LoF and D-Mis variants to identify genes potentially related to susceptibility in ASD patients. Genes showing a nominally significant association are described in Tables S4 and S5. Although no genes reached statistical significance following multiple testing correction, we found a nominally significant association (*P* = 0.043) with ASD for LoF variants in *ABCA13*, which is known to be related to synaptic vesicle endocytosis [34] and reported as an ASD candidate gene in the SFARI database. All of the rare *ABCA13* variants were LoF variants and not registered in the gnomAD database for the East Asian population. We validated all LoF variants by Sanger sequencing, and the variant information and clinical phenotypes is described in Fig. S5 and Table 2. Three of five carriers of *ABCA13* LoF variants had symptoms of attention deficit hyperactivity disorder (ADHD).

**Table 2:** Phenotypes of ASD carriers with rare variants in *ABCA13*.

Sample ID/Sex/Age	Position	Base change	Protein variant and variant effect	Inheritance	Congenital abnormality	ID (IQ < 70)	ADHD	Tic disorders	Motor delay	Epileptic seizure	Sensory hypersensitivity	Mood disorders	OCD	Psychotic symptons	Detailed phenotype
		M > m													
228/M/9	7:48259087	C > T	p.Arg142*: stop_gained	inherited (maternal)	−	−	−	−	−	−	+	−	−	−	Language delay, poor emotional control
623/F/25	7:48280581	G > T	p.E394*: stop_gained	unknown	−	+	−	−	−	−	−	−	−	+	Mild ID
209/F/6	7:48318518	TA > T	p.I2579*: frameshift_variant	inherited (paternal)	−	−	+	−	−	−	+	−	−	−	Motor coordination deficits
239/M/9	7:48319055	G > A	p.W2765*: stop_gained	inherited (paternal)	−	−	+	−	−	−	+	−	−	−	Night terrors, bedwetting
455M/41	7:48392085	G > C	splice_donor_variant	unknown	−	−	+	−	−	−	−	+	−	−	Attempted suicide and depressive episodes

Note: The variants in this table were validated by Sanger sequencing. Positions of allele/amino acid changes in *ABCA13* were determined with reference to the following ensemble transcription ID based on NCBI Build GRCh37/hg19: ENST00000435803. *M* major allele, *m* minor allele, *MAC* minor allele count, *MAF* minor allele frequency, *ID* intellectual disorder, *ADHD* attention deficit hyperactivity disorder, *OCD* obsessive compulsive disorder.

## Discussion

To the best of our knowledge, this is the largest case-control WES study of Japanese ASD patients. Using a case-control study approach, we were able to capture a broader range of rare variants (including rare inherited variants and *de novo* rare variants) compared with a previous study of rare *de novo* variants in trios [14]. In this study, we performed rare variant burden tests and demonstrated the enrichment of rare variants in constrained genes, FMRP target genes [35, 36], and synaptic function-related genes in Japanese ASD. Although, these findings are similar to functional categories that have been implicated in samples of European ancestry [37], our gene-based burden test data strengthen the evidence regarding *ABCA13*, which is related to synaptic vesicle endocytosis. Although this study included some ASD samples included in the previous study of rare *de novo* variants in trios, all of the LoF variants in *ABCA13* identified in this study were rare inherited variants that were not detected in the previous study.

A burden test with GO terms focusing on synaptic function revealed that rare damaging variants (LoF and D-Mis variants) were enriched in trans-synaptic signaling (GO:0099537). Genes related to trans-synaptic signaling include several known ASD susceptibility genes [38–40] that affect neuronal connectivity in the brain by decreasing or increasing synapse strength and number [41]. Among the trans-synaptic signaling–related genes identified in this study, we discovered several possible ASD candidate genes, such as *PTPRD* and *AKAP7*, for which deleterious variants were repeatedly detected only in ASD cases in this study. PTPRD, a receptor protein tyrosine phosphatase genetically associated with neurodevelopmental disorders, regulates receptor tyrosine kinases to ensure appropriate numbers of neurons [42–44]. AKAP7 regulates signaling cascades downstream of D1-like dopamine receptors, and it is suggested to regulate two ASD-implicated biological processes (innate immunity and melatonin synthesis) for which new treatments have been proposed through modulating the downstream effects of risperidone treatment in ASD patients [45]. Interestingly, via the expression analysis, we demonstrated that genes in trans-synaptic signaling detected in Japanese ASD patients are enriched during the early mid-fetal period in the cortex and enriched in cortical neurons, which has also been implicated in the pathogenesis of ASD in analyses of samples of European ancestry [46]. In contrast, no enrichment was observed in the gene set related to the chromatin function, which has also been linked with the pathophysiology of ASD susceptibility [37, 40, 41]. We also could not demonstrate any enrichment of ASD candidate genes in the SFARI database. These negative results are probably due to the sample size.

Furthermore, among genes exhibiting a nominally significant association by gene-based burden analysis in this study, *ABCA13*, which is not a SynGO-related gene, could be an ASD candidate gene because it has been linked to synapse function [34, 47]. *ABCA13* encodes ATP-binding cassette (ABC) subfamily A member 13, a transmembrane protein with the typical ABC protein structure, and it has been suggested as playing an important role in accelerating synaptic vesicular endocytosis in cortical neurons [34]. Interestingly, a monkey carrying a heterozygous *ABCA13* deletion exhibited impaired social ability and restricted and repetitive behaviors that are commonly associated with ASD [48]. From the viewpoint of human genetics studies, although *ABCA13* harbors a relatively large number of LoF variants (pLI = 0), researchers have suggested that SNVs in *ABCA13* confer susceptibility to neuropsychiatric disorders, including schizophrenia [49] and ASD [40]. Therefore, accumulating evidence, including data from this study, supports *ABCA13* as an ASD candidate gene. It is of note that three of five ASD patients carrying the ABCA13 mutations have comorbid ADHD. ABCA13 plays a role in synapse traffic endocytosis that is thought to alter neuronal activity and neurotransmitter release [34], which could contribute to the pathophysiology of ADHD [50]. Furthermore, a recent WGS study of 205 ADHD patients identified *ABCA13* as an ADHD candidate gene based on the finding of two frameshift variants in *ABCA13* [51].

Our study has several limitations. First, our sample size was relatively small. Although the sample size was small compared with a reported sequencing study using samples of European ancestry [11], it is of note that the Japanese population is considered genetically homogeneous [52], which can be beneficial in sequencing studies due to decreased allelic diversity [53–55]. Second, our WES data did not cover several potentially informative regions, including untranslated regions and intronic regions. Recent studies have reported that intronic/intergenic variants that affect expression regulation identified by WGS contribute more to the genetic risk of ASD than exonic variants [56]. Furthermore, a recent single-cell analysis of gene expression and chromatin accessibility revealed the mechanisms of neurodevelopment in detail [57]. Therefore, in future studies, WGS would be useful in performing more refined evaluations of brain development in relation to ASD because the expression analysis of the present study was based only on rare variants in the exon regions. Third, we could not fully conduct phenotypic analyses because we could not obtain clinical information during the developmental period.

Overall, this study, involving the largest case-control WES analysis of Japanese ASD patients, demonstrated that ASD candidate rare variants are primarily involved in synaptic function. In particular, we strengthen the evidence regarding the role of *ABCA13*, a synaptic function-related gene, in Japanese ASD pathobiology. In future studies, it would be useful to expand the sample size by aggregating WGS/WES data through collaborations within Japan to characterize Japanese ASD-specific pathophysiology. Furthermore, combined analyses also involving non-Japanese samples would be useful as a means of evaluating the trans-ethnic generalizability of our results.

## Supplementary information

Supple

## Data Availability

We performed this study using WES data of Japanese ASD patients from three sequencing sites: The Broad Institute (ASD = 256, HCs = 299), Yokohama City University (ASD = 51), and Nagoya University (ASD = 2). Detailed information regarding data availability are provided in dbGaP under study accession number phs000298.v4.p3 [19] and the Human Genetic Variation database under accession number HGV0000007 [14]. Combined variants data used in this study are available from the corresponding author upon request (branko@med.nagoya-u.ac.jp).
