# Supplementary material for: Exome sequencing analysis of Japanese autism spectrum disorder case-control sample supports an increased burden of synaptic function-related genes
Source: Transl Psychiatry. 2022 Jul 11;12:265. doi: 10.1038/s41398-022-02033-6 (PMC9271461; doi:10.1038/s41398-022-02033-6)
Supplement: Supplementary file 1 — Supple [file 41398_2022_2033_MOESM1_ESM.docx]

**Supplementary Information**

**Title:** **Exome sequencing analysis of Japanese autism spectrum disorder case-control sample supports an increased burden of synaptic function–related genes**

Hiroki Kimura^1)^, Masahiro Nakatochi^2)^, Branko Aleksic^1)*^, James Guevara^3)^, Miho Toyama^1)^, Yu Hayashi^1)^, Hidekazu Kato^1)^, Itaru Kushima^1)4)^, Mako Morikawa^1)^, Kanako Ishizuka^５)^, Takashi Okada^６)^, Yoshinori Tsurusaki^7)8)^, Atsushi Fujita^７)^, Noriko Miyake^７) ９)^, Tomoo Ogi^１０)^, Atsushi Takata^７) １１)^, Naomichi Matsumoto^７)^, Joseph Buxbaum^1２)^, Norio Ozaki^1) 13)**^ and Jonathan Sebat^3)**^

Affiliations:

1. Department of Psychiatry, Nagoya University Graduate School of Medicine, Nagoya, Aichi, Japan
2. Public Health Informatics Unit, Department of Integrated Health Sciences, Nagoya University Graduate School of Medicine, Nagoya, Aichi, Japan
3. Department of Psychiatry, University of California San Diego, CA, USA
4. Medical Genomics Center, Nagoya University Hospital, Aichi, Japan
5. Health Support Center, Nagoya Institute of Technology, Aichi, Japan
6. Department of Developmental Disorders, National Institute of Mental Health,

National Center of Neurology and Psychiatry, Tokyo, Japan

1. Department of Human Genetics, Yokohama City University Graduate School of Medicine, Kanagawa, Japan
2. Faculty of Nutritional Science, Sagami Women’s University, Sagamihara, Japan
3. Department of Human Genetics, National Center for Global Health and Medicine, Tokyo, Japan
4. Department of Genetics, Research Institute of Environmental Medicine, Nagoya University, Aichi, Japan; Department of Human Genetics and Molecular Biology, Nagoya University Graduate School of Medicine, Aichi, Japan.
5. Laboratory for Molecular Pathology of Psychiatric Disorders, RIKEN Center for Brain Science, Wako, Saitama, Japan
6. Department of Psychiatry, Mount Sinai University, NY, USA
7. Institute for Glyco-core Research (iGCORE), Nagoya University, Chikusa-ku, Nagoya, Japan

*Corresponding author

**Co-last author

Corresponding author:

Branko Aleksic, MD, PhD Associate Professor

Department of Psychiatry, Nagoya University Graduate School of Medicine

65 Tsurumai-cho, Showa-ku, Nagoya, Aichi 466-8550, Japan

Tel: +81 52 7442282; Fax: +81 52 7442293

E-mail: [branko@med.nagoya-u.ac.jp](mailto:branko@med.nagoya-u.ac.jp)

**The contents of supplementary materials**

**Figure S1. Primary filtering steps in this study.**

**Figure S2. Evaluation of principal components for each sample.**

**Figure S3. Count of rare variants per individual.**

**Figure S4. Visualization of SynGO analysis regarding cellular component.**

**Figure S5. Locations of prioritized rare variants in *ABCA13*.**

**Table S1. Details of samples analyzed in this study.**

**Table S2. Results of transcriptomic analyses to identify brain regions and periods associated with ASD.**

**Table S3. Result of transcriptomic analyses to identify cell types associated with ASD.**

**Table S4. Genes nominally associated with ASD as determined by gene-based burden analysis using LoF and D-mis variants.**

**Table S5. Genes nominally associated with ASD as determined by gene-based burden analysis using LoF variants.**

**Figure S1. Primary filtering steps used in this study.**

**
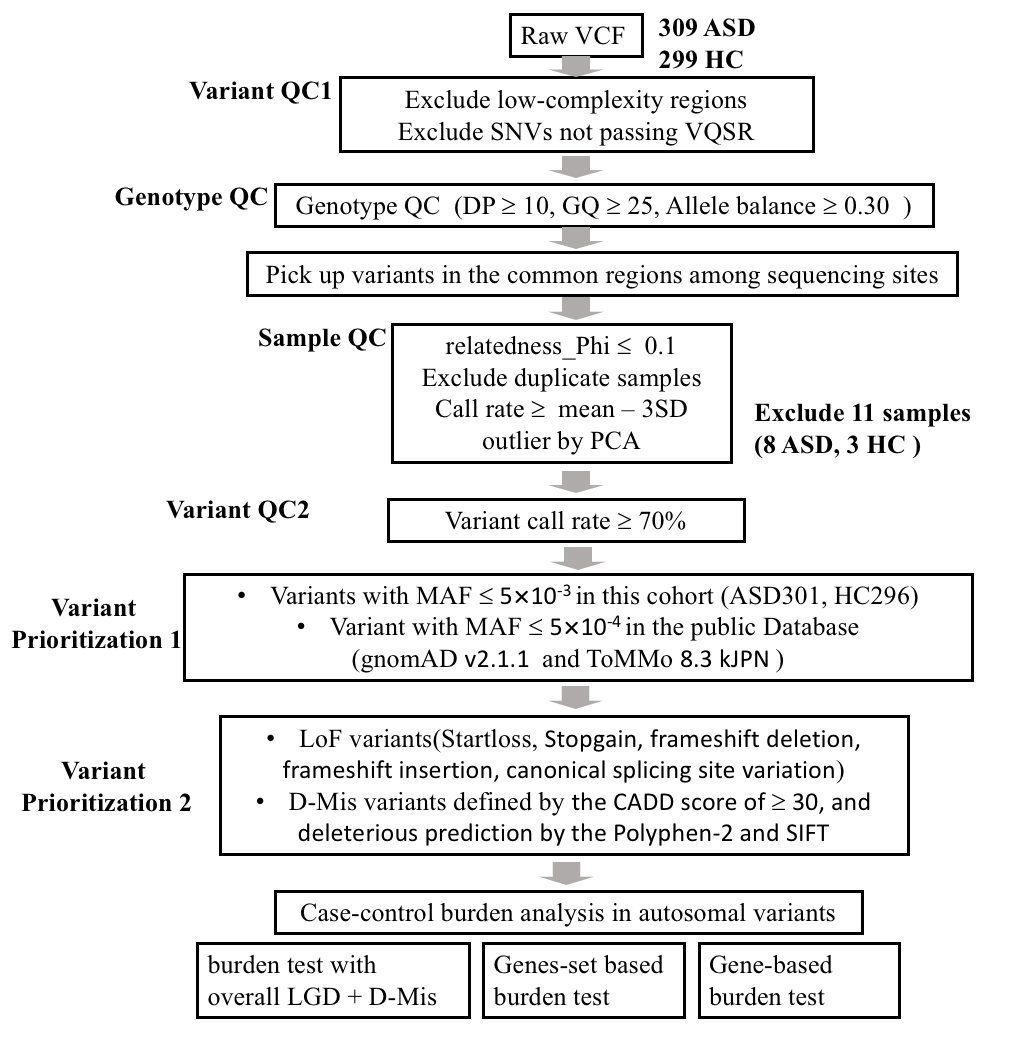
**

**Legend.** This workflow shows the primary filtering steps employed to pick prioritized rare variants that could be associated with ASD susceptibility. For the QC, we first excluded variants in low-complexity regions and variants that did not pass the variant quality score recalibration (VQSR) filtering. For individual-level genotype QC, genotype calls with read depth < 10, genotype quality < 25, and allele balance < 30 were excluded. We picked variants in the common regions sequenced by each platform used in this study. Furthermore, we removed the following condition samples: 1) samples from the same family, 2) duplicate samples, 3) low-quality samples (call rate < mean – 3SD), 4) outlier samples as determined by PCA. We then filtered variants with a call rate < 70% across all samples. we used the following procedure to analyze only autosomal rare variants. The variants with a MAF > 5.0 ×10^−4^ were excluded using the following public database: The Genome Aggregation Database (gnomAD V2.1.1) and Japanese Multi Omics Reference Panel (jMorp ToMMo 8.3 kJPN v20200831) (<https://jmorp.megabank.tohoku.ac.jp/202102/>). Furthermore, variants with a MAF > 5.0 ×10^−3^ in our case-control data were excluded. The overall number of rare variants for each sample was calculated and used as a confounding variable in the subsequent burden test. Each individual’s overall number of detected rare variants is shown in Figure S3. To prioritize variants for ASD pathophysiology, we picked likely loss of function (LoF) variants (startloss, stopgain, stoploss, frameshift deletion, frameshift insertion, canonical splicing site variation), and putative deleterious missense variants (D-mis) defined according to the following condition: Combined Annotation-Dependent Depletion (CADD) score ≥ 30 and deleterious prediction by Polyphen-2 and SIFT. These prioritized variant sets were then used as the starting point for rare variant case-control association testing. These prioritized variants set were then used for rare variant case-control burden analysis.

**Figure S2. Results of principal component analysis.**

**
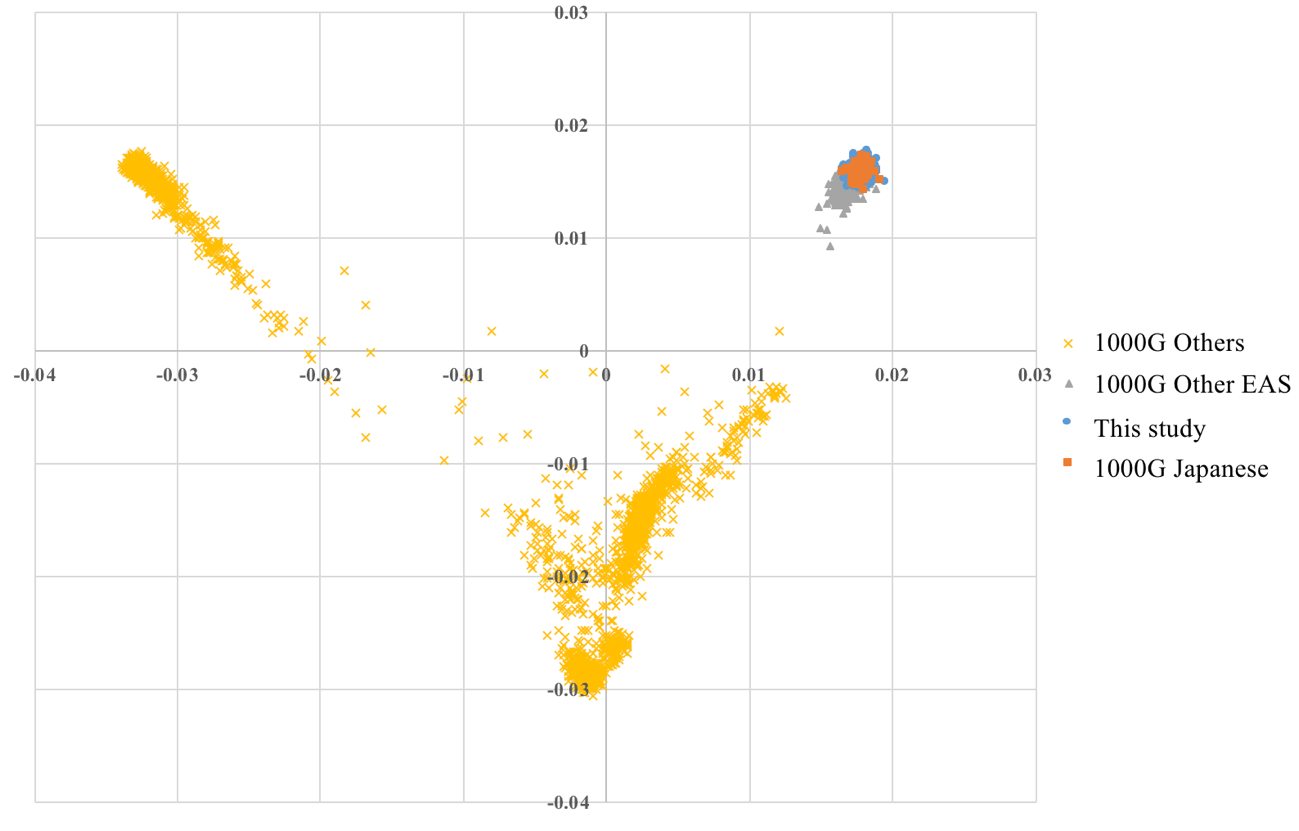
**

**Legend.** Principal component analysis (PCA) was performed to exclude population outliers. PCA with the 1000 Genomes Project reference panel (phase 3) detected no subject with probable ancestries outside of the east Asian population and ensured that all probands seemed to be Japanese origin. Red square, gray triangle and orange cross indicate Japanese individuals, other East Asian (EAS) individuals, and the others of the 1000G, respectively. Blue circles indicate 301 ASD proband and 296 control exomes in this study.

**Figure S3. Count of rare variants per individual.**

**
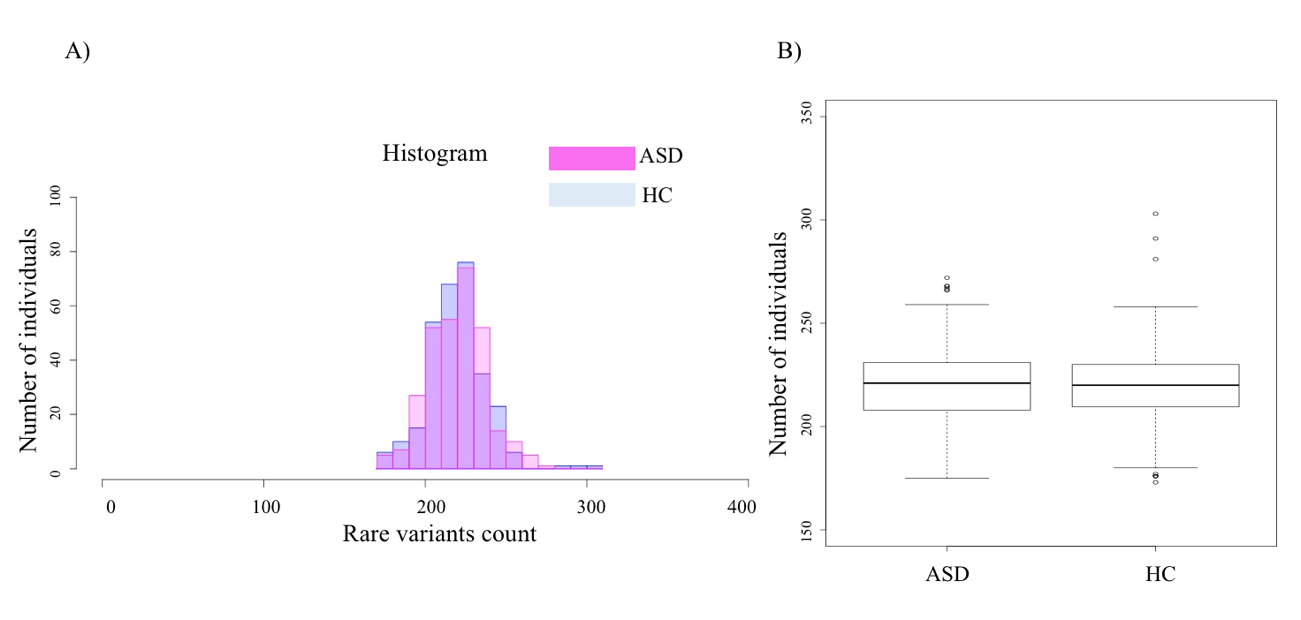
**

**Legend.** A) Histogram of per-individual rare variants (X-axis) and the number of individuals with the corresponding rare variants (Y-axis) among the 302 ASD cases and 299 controls. The number of rare variants was picked after variant prioritization 1 (Figure S1). B) Boxplot: box represents the middle 50% of observations. Middle bold line represents the median gene expression. Whiskers represent the minimum and maximum observations. The difference between each number of rare variants among ASD and HC samples was not statistically significant (P = 0.51 by Wilcoxon rank sum test).

**Figure S4. Visualization of SynGO analysis regarding cellular component.**


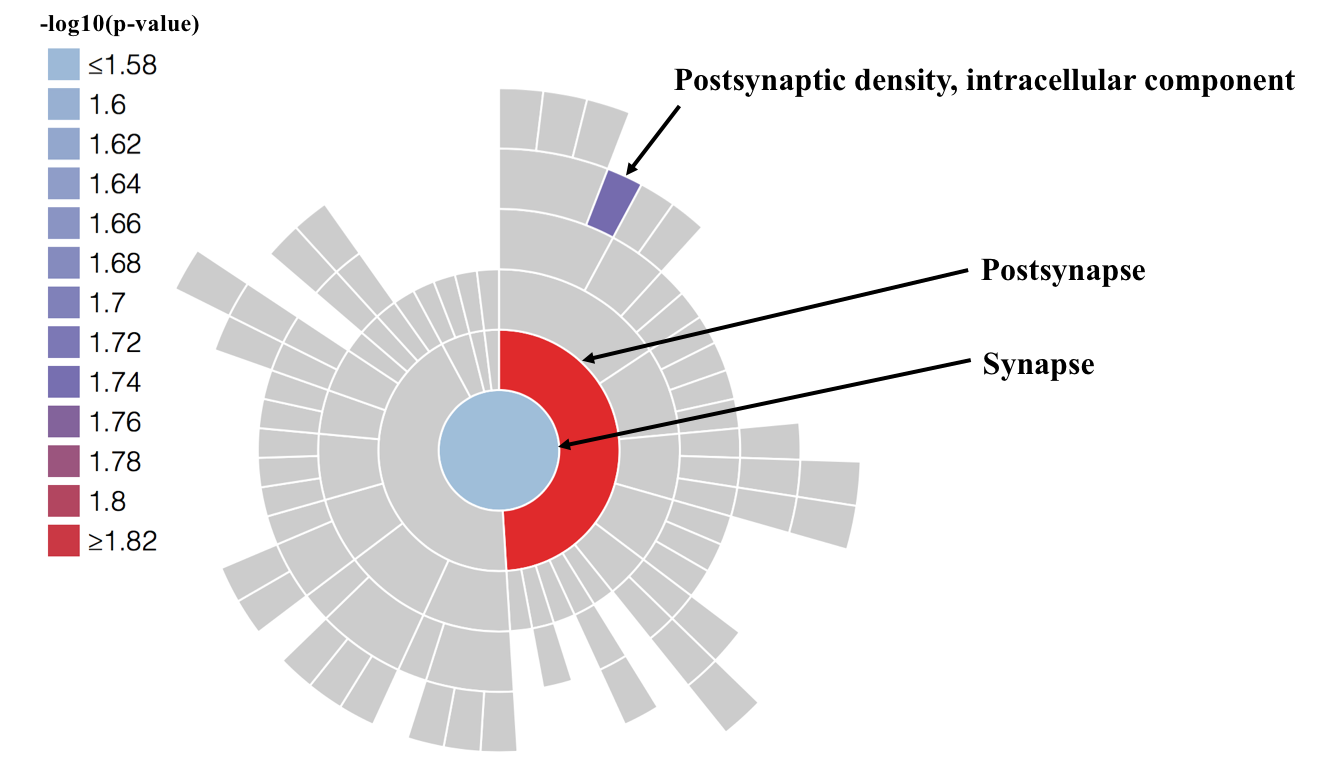


**Legend.** SynGO terms were visualized using Custom color-coding of SynGO ontologies (<https://www.syngoportal.org/plotter.html>). To visualize this ontology hierarchy and provide a standardized visualization of SynGO annotations, all cellular component (CC) terms populated with gene annotations in SynGO were plotted in a circular fashion, with the highest hierarchical term (synapse) in the center and each layer of subclasses in outward concentric rings. We then colored ontology terms with P-values < 0.05 from the Results of burden test with gene set in SynGO (Table 1). In the cellular component category, post-synapse (P = 0.014, Q-value = 0.21) and post-synaptic density, intracellular component (P = 0.018, Q-value = 0.20) (a subclass of post-synapse), showed a nominally significant association with ASD.

**Figure S5. Locations of prioritized rare variants in *ABCA13*.**

**
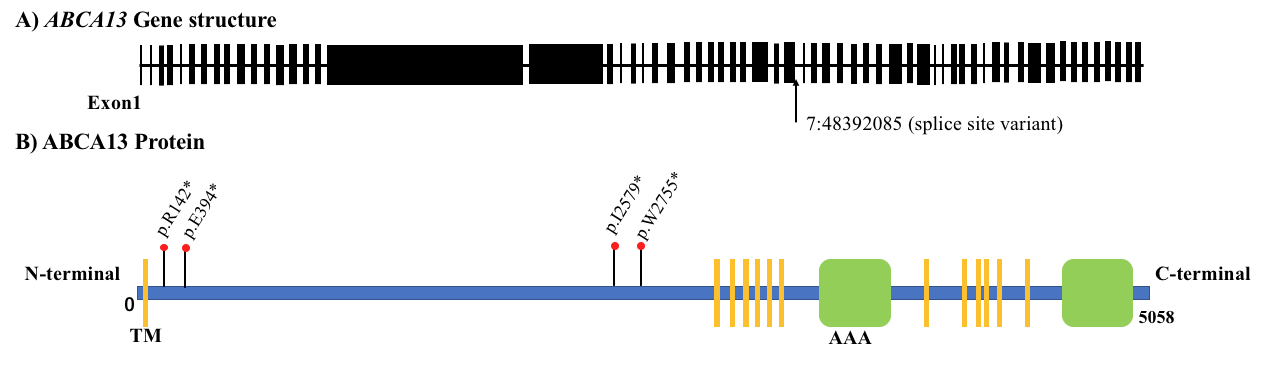
**

**Legend.** (A) *ABCA13* gene structure based on ENST00000435803.1; black boxes indicate the protein-coding exons sequenced in this study. We discovered a canonical splice site variant 1 bp downstream of exon 31 (7:48392085). (B) ABCA13 protein structure (5058 amino acids); three stop-gain variants and a frameshift variant are shown in red outside the known functional domains as determined based on the Human Protein Reference Database (<http://www.hprd.org>). TM, transmembrane domain; AAA, ATPases associated with a variety of cellular activities.

**Table S1. Details of samples analyzed in this study.**

**Note.** BI; Broad Institute, YCU; Yokohama City University, NU; Nagoya University, ASD; Autism spectrum disorder, HC; Healthy Control. The detailed descriptions of each sequencing method were referred to each report(1-3).

**Table S2. Results of transcriptomic analysis to identify brain regions and period associated with ASD.**

**Note.** We performed specific expression analyses across brain regions and development using the genes associated with trans-synaptic signaling detected in ASD cases of this study. The first row lists the pSI (specificity index) (4) thresholds, and the first column lists the adult brain regions and period of development. Values in the table are Fisher's exact P-values, followed by the Benjamini-Hochberg corrected values in parentheses.

**Table S3. Results of transcriptomic analysis to identify cell types associated with ASD.**

**Note.** We performed cell type–specific expression analysis of genes associated with trans-synaptic signaling detected in ASD cases of this study. The first row lists the pSI (specificity index) (4) thresholds, and the first column lists brain cell types. Values in the table are Fisher's exact P-values, followed by the Benjamini-Hochberg corrected values in parentheses.

**Table S4. Genes nominally associated with ASD by LoF and D-mis variants**

**Note.** MAC_Burden, number of allele counts used for the burden analysis; mean_case, mean number of variants in one case; mean_ctrl, mean number of variants in one healthy control.

**Table S5. Genes nominally associated with ASD as determined by gene-based burden analysis using LoF variants.**

**Note.** MAC_Burden, number of allele counts used for the burden analysis; mean_case, mean number of variants in one case; mean_ctrl, mean number of variants in one healthy control.

**Reference**

1. Takata A, Miyake N, Tsurusaki Y, Fukai R, Miyatake S, Koshimizu E, et al. Integrative Analyses of De Novo Mutations Provide Deeper Biological Insights into Autism Spectrum Disorder. Cell Rep. 2018;22(3):734-47.

2. Satterstrom FK, Kosmicki JA, Wang J, Breen MS, De Rubeis S, An JY, et al. Large-Scale Exome Sequencing Study Implicates Both Developmental and Functional Changes in the Neurobiology of Autism. Cell. 2020;180(3):568-84 e23.

3. Oka Y, Hamada M, Nakazawa Y, Muramatsu H, Okuno Y, Higasa K, et al. Digenic mutations in ALDH2 and ADH5 impair formaldehyde clearance and cause a multisystem disorder, AMeD syndrome. Sci Adv. 2020;6(51).

4. Dougherty JD, Schmidt EF, Nakajima M, Heintz N. Analytical approaches to RNA profiling data for the identification of genes enriched in specific cells. Nucleic Acids Res. 2010;38(13):4218-30.
